# NFIB Regulates Chemoresistance in Small Cell Lung Cancer by Suppressing Notch Signaling Activity

**DOI:** 10.1002/cnr2.70526

**Published:** 2026-04-19

**Authors:** Weixin Qin, Ziyan Wang, Shuzhe Deng, Huilei Qiu, Hongxue Meng, Jingshu Geng

**Affiliations:** ^1^ Department of Pathology Harbin Medical University Cancer Hospital, Harbin Medical University Harbin Hei Longjiang China

**Keywords:** chemoresistance, heterogeneity, NFIB, notch, SCLC

## Abstract

**Background:**

Small cell lung cancer (SCLC) is an aggressive malignancy characterized by the rapid development of therapy resistance, the underlying mechanisms of which remain incompletely understood. The transcription factor NFIB is a recognized oncogene in SCLC, promoting tumor progression by regulating metastasis and proliferation. However, its potential role in mediating chemotherapy resistance is poorly defined.

**Aims:**

This study aimed to elucidate the mechanism by which NFIB regulates chemoresistance in SCLC and to assess the therapeutic potential of co‐targeting NFIB and the Notch signaling pathway.

**Methods and Results:**

Expression of NFIB and associated genes was analyzed in SCLC cell lines and clinical samples using Western blotting, quantitative real‐time PCR (qRT‐PCR), immunofluorescence (IF), and immunohistochemistry (IHC). Transcriptional regulation was examined by chromatin immunoprecipitation (ChIP), and drug sensitivity was measured via CCK‐8 assays. We found that NFIB acts as a dual‐function regulator, driving oncogenesis and controlling chemoresistance. NFIB knockdown activated the endogenous Notch pathway, which in turn promoted drug resistance. NFIB expression positively correlated with neuroendocrine (NE) markers and contributed to tumor heterogeneity, a process modulated by Notch1. Mechanistically, loss of NFIB relieved its repression of Notch1, leading to suppressed NE gene expression and yielding slow‐cycling, chemoresistant cells with activated Notch signaling. Critically, combining Notch pathway inhibition with chemotherapy attenuated intratumoral heterogeneity and reversed this resistance phenotype.

**Conclusions:**

NFIB plays a paradoxical role in SCLC, serving as both an oncogenic driver and a key regulator of chemoresistance via Notch pathway activation. These findings revealed a novel resistance mechanism and propose a promising therapeutic strategy of combined Notch inhibition and chemotherapy for SCLC treatment.

## 1 Introduction

Small cell lung cancer (SCLC) comprises approximately 13% of all lung cancer cases and poses a formidable clinical challenge due to its rapid proliferation and propensity for early metastasis [1]. Although patients typically exhibit initial chemosensitivity, they almost invariably develop rapid chemoresistance, resulting in a dismal five‐year overall survival (OS) rate of less than 10% [2]. Over the past three decades, advances in first‐line therapies for SCLC have been marginal, and the development of effective targeted agents remains strikingly limited [3]. Despite extensive research showing SCLC is an aggressive malignancy with limited therapeutic options, the underlying molecular mechanisms driving its resistance remain poorly understood.

The Notch signaling pathway is essential for cell fate determination in diverse tissues [4]. During lung development, activation of Notch family members potently suppresses intrinsic NE differentiation [5, 6, 7]. In the context of SCLC, however, Notch signaling can exert pro‐tumorigenic functions by promoting the expansion of non‐neuroendocrine (non‐NE) cell populations [8, 9, 10, 11, 12]. This functional duality is further underscored by the pathway's ability to reprogram a subset of SCLC cells, driving a phenotypic shift from NE to non‐NE states [13]. Among Notch1‐regulated genes, Hes1—a key helix–loop–helix transcription factor—has been linked to SCLC progression and poorer patient prognosis. Notably, ASCL1, the master transcriptional regulator of NE differentiation programs, has been identified as a direct downstream target of Notch signaling. This finding reveals a coherent regulatory circuit in which Notch‐mediated repression of ASCL1 disrupts NE lineage commitment, thereby orchestrating pivotal oncogenic processes during SCLC tumorigenesis [14].

NFIB is a member of the nuclear factor I (NFI) family of transcription factors, which play critical roles in neurodevelopment and lung morphogenesis [15, 16]. This family comprises four distinct genes—NFIA, NFIB, NFIC, and NFIX—all of which encode a conserved DNA‐binding domain and are involved in adenoviral DNA replication as well as the transcriptional regulation of diverse cellular and viral genes [17]. Importantly, NFIB has been established as an oncogene in SCLC [18, 19]. In SCLC mouse models, NFIB promotes metastasis by orchestrating global chromatin remodeling and reprogramming the metastatic transcriptome, with its overexpression linked to increased chromatin accessibility [20, 21]. While NFIB upregulation is recognized as pivotal for SCLC tumor development and metastasis, its specific contribution to chemoresistance remains uncharacterized. Therefore, the aim of this study is to systematically investigate the role of NFIB in mediating chemoresistance in SCLC.

## 2 Materials and Methods

### 2.1 Cell Culture and Transfection

Human cell lines (HBE, SHP77, H2227, H524, H69, H209, and H446) were purchased from ATCC and maintained in RPMI‐1640 medium supplemented with 10% fetal bovine serum (FBS) and 1% penicillin–streptomycin–glutamine at 37°C in a humidified 5% CO_2_ atmosphere. Lentiviral constructs for NFIB knockdown (shRNA), NFIB overexpression, and Notch1 intracellular domain (N1ICD) expression were designed and validated by GeneChem (Shanghai, China). Cells were infected with lentivirus for 12 h, followed by puromycin selection for 14 days to generate stable pools. Knockdown and overexpression efficiencies were confirmed by qRT‐PCR and Western blot analysis. The nomenclature and corresponding treatment conditions for the cell lines are provided in Table S3.

### 2.2 Western Blotting

Cells were lysed in ice‐cold lysis buffer (50 mM Tris–HCl, pH 7.5; 150 mM NaCl; 0.1% SDS; 1% Triton X‐100; 0.5% sodium deoxycholate; 5 mM EDTA) supplemented with protease and phosphatase inhibitors. Protein concentration was determined using a BCA assay. Equal amounts of protein were denatured in 1× Laemmli buffer, separated on 4%–20% SDS‐PAGE gels, and subsequently transferred onto nitrocellulose membranes. To ensure equal loading, protein levels were normalized to GAPDH or β‐actin. A complete list of antibodies is provided in Table S1.

### 2.3 Quantitative Real‐Time PCR (qRT‐PCR)

Total RNA was extracted using TRIzol reagent (Invitrogen) according to the manufacturer's instructions. cDNA was synthesized from equal amounts of RNA using the Transcriptor First Strand cDNA Synthesis Kit (Roche). Quantitative PCR was performed on a LightCycler 480 System (Roche). Gene expression levels were normalized to GAPDH, and all primer sequences are listed in Table S2.

### 2.4 Cell Viability and Apoptosis Assays

For viability assays, cells were seeded at a density of 3000 cells/well in 96‐well plates and treated with the indicated compounds at designated concentrations. Cell viability was assessed using a Cell Counting Kit‐8 (CCK‐8; Beyotime, China). After incubating with CCK‐8 reagent for 2 h at 37°C, absorbance was measured at 450 nm using a Tecan Sunrise microplate reader. Dose–response curves were generated by non‐linear regression in GraphPad Prism 8.0, and the half‐maximal inhibitory concentration (IC50) was calculated accordingly.

For apoptosis analysis, 1 × 10^6^ cells were harvested, washed with cold PBS, and resuspended in binding buffer. Cells were then stained using an Annexin V‐FITC/PI Apoptosis Detection Kit (BD Biosciences, 556 547) and analyzed by flow cytometry. Apoptosis‐related proteins (BIK, BAK, BAX, BCL‐2) were detected by Western blotting as described in Section 2.

### 2.5 Drug Treatments

Cells were seeded at 3000 cells per well in 96‐well plates. After 24 h, they were treated with the following compounds at the indicated concentrations: cisplatin (Teva; 0.1–20 μM), dibenzazepine (DBZ; Selleckchem, S2711; 10 μM), and tarextumab (OMP‐59R5; 100 μg/mL). Cell viability was assessed 24 h post‐treatment using a CCK‐8 assay as described in Section 4.

### 2.6 Chromatin Immunoprecipitation and Binding Site Analysis

Chromatin immunoprecipitation was performed using the SimpleChIP Enzymatic Chromatin IP Kit (CST, #9003) according to the manufacturer's protocol with modifications [22]. Briefly, cells were crosslinked with 1% formaldehyde for 10 min at room temperature, and the reaction was quenched with 0.125 M glycine. Cells were then washed and lysed. Chromatin was digested with Micrococcal Nuclease (0.5 μL per sample; NEB #10011) at 37°C for 20 min, followed by sonication to shear DNA to 300–500 bp fragments. After pre‐clearing with protein A agarose beads, chromatin (20–30 μg) was immunoprecipitated overnight at 4°C using 2 μg of anti‐NFIB antibody (Abcam; ab186738) or 10 μg of normal IgG as a control. Immune complexes were washed, eluted, and treated with RNase and proteinase K. Crosslinks were reversed by incubation at 65°C overnight, and DNA was purified by phenol‐chloroform extraction and ethanol precipitation.

Purified ChIP DNA was analyzed by qPCR using SYBR Green Supermix (Bio‐Rad) on a CFX Connect Real‐Time PCR system, with primers listed in Table S2. Putative NFIB‐binding sites in the promoters of NOTCH1, BAK, and BIK were predicted in silico using the JASPAR database (2024 release; profile MA1643.1). The scanned genomic regions were: NOTCH1 (chr9:136492433–136 494 433), BAK (chr22:43110651–43 112 750), and BIK (chr6:33572453–33 574 552). Predictions were filtered using a relative profile score threshold of 85% and visualized with the built‐in JASPAR function.

### 2.7 Luciferase Reporter Assay

A luciferase reporter plasmid was generated by inserting the human NOTCH1 promoter region (−1105 to +15 bp) into the pGL3‐Basic vector. Putative NFIB‐binding sites within this promoter were mutated using the QuikChange II XL Site‐Directed Mutagenesis Kit with the following primers: Mut1, 5′‐AGGGAGGCACGAGGCCCACTT‐3′; Mut2, 5′‐TCCCTGGCACGAGGCCCACTT‐3′. All constructs were verified by sequencing. For the assay, cells were co‐transfected with the pGL3‐promoter plasmid and a pCMV‐*β*‐galactosidase control plasmid using Lipofectamine 2000. After 24 h, luciferase activity was measured with a Luciferase Assay System, and *β*‐galactosidase activity was quantified with the Galacto‐Star Chemiluminescent Assay on an EnVision multilabel reader. Reporter activity was normalized to the *β*‐galactosidase internal control.

### 2.8 Xenograft Mouse Experiment

Five‐week‐old male BALB/c nude mice were subcutaneously inoculated with NFIB‐knockdown or control SCLC cells (*n* = 5 per group). Three weeks post‐inoculation, mice were randomized into six treatment groups using a computer‐generated block design, stratified by baseline tumor volume. Groups received intraperitoneal injections every 48 h as follows: cisplatin (4 mg/kg), etoposide (4 mg/kg), tarextumab (20 mg/kg), cisplatin + etoposide (both 4 mg/kg), cisplatin + etoposide + tarextumab, or PBS vehicle. Mice were euthanized 40 days post‐inoculation. Tumors were excised, weighed, and measured; volume was calculated as (length × width)^2^/2. Excised tissues were processed for immunohistochemical analysis of NFIB, Hes1, and Notch1 expression.

### 2.9 Immunofluorescence (IF) and Immunohistochemistry (IHC)

For IF staining, cells grown on glass coverslips were fixed with 4% paraformaldehyde, permeabilized with 0.25% Triton X‐100, and blocked with serum. They were then incubated with primary antibodies overnight at 4°C, followed by species‐matched fluorescent secondary antibodies. Nuclei were counterstained with DAPI, and coverslips were mounted for imaging. Images were captured using a Leica DM3000 inverted fluorescence microscope.

IHC staining was performed on formalin‐fixed, paraffin‐embedded tissue sections as previously described [11].

### 2.10 Statistical Analysis

Data are presented as mean ± SEM from at least three independent biological replicates. Statistical significance was determined using Student's *t*‐test (for two‐group comparisons) or one‐way ANOVA followed by appropriate post hoc tests (for multiple groups). For data that did not meet assumptions of normality or equal variance, non‐parametric tests (e.g., Kruskal–Wallis test) were applied. All analyses were performed using GraphPad Prism (version 8.0) or SPSS (version 25), and a *p* value < 0.05 was considered statistically significant.

## 3 Results

### 3.1 NFIB Expression Positively Correlates With Proliferation, Survival, and Metastasis in SCLC

**FIGURE 1:**
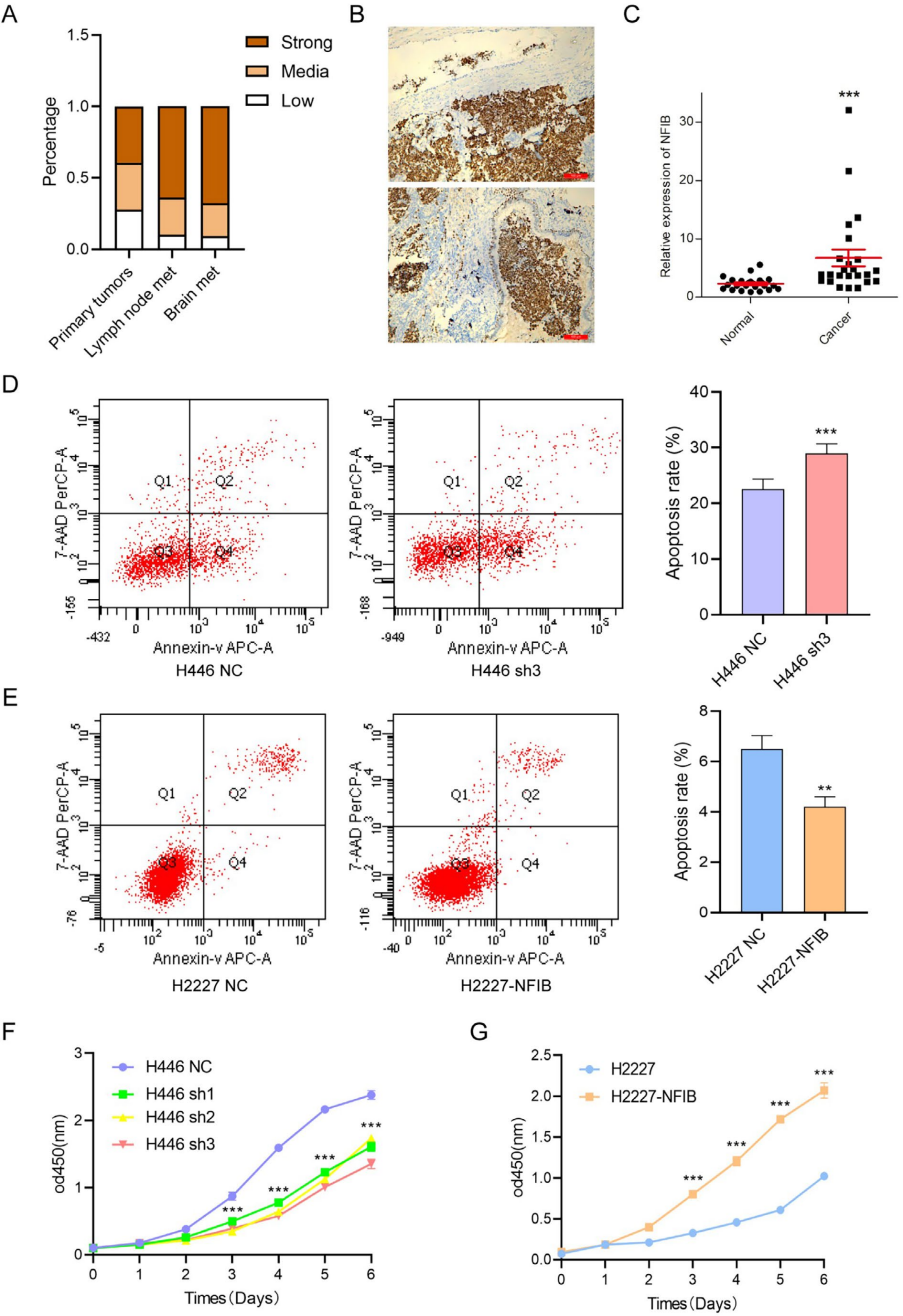
NFIB suppresses apoptosis and promotes proliferation in SCLC. (A) Quantification of NFIB expression detected by IHC in human SCLC samples, including primary tumors without metastasis (*n* = 61), primary tumors with lung lymph node metastases (*n* = 59), and brain metastases (*n* = 65). IHC scoring criteria: Low/negative, 0–3; Medium, 4–8; High, 9–12. (B) NFIB staining in bronchiolar and vascular spaces of the lung, indicating intrapulmonary metastatic lesions. (C) qRT‐PCR analysis of NFIB expression levels was performed using matched tumor and paracancerous tissue pairs from SCLC patients. (D and E) Flow cytometry and PI/Annexin V staining were performed in SCLC cells after NFIB knockdown (D) or overexpression (E). (F and G) Growth curves of SCLC cells following NFIB knockdown (F) or overexpression (G), as assessed by CCK‐8 assay. Representative results from three independent experiments performed in triplicate are presented as mean ± SEM. ***p < 0.001, by *t* test.

Previous experiments have confirmed that NFIB is an oncogene in SCLC and that it accelerates SCLC initiation and progression [18, 19, 20, 21]. To further characterize its expression profile and association with tumor progression, we first examined NFIB levels in clinical specimens and cell lines. Analysis of Clinical Specimens: Immunohistochemical analysis revealed NFIB immunopositivity in 80.7% (96/119) of primary SCLC tumors and 90.8% (59/65) of brain metastatic lesions, whereas carcinoid and large‐cell neuroendocrine carcinoma (LCNEC) tissues showed negligible expression (Figure 1A,B; Figure S1). NFIB positivity was significantly higher in brain metastases compared to primary tumors. Elevated NFIB expression correlated strongly with adverse clinicopathological features, including regional lymph node metastasis, distant metastasis, advanced TNM stage (Table 1), as well as intrapulmonary metastasis and vascular invasion (Figure 1B). Importantly, high NFIB levels were negatively associated with chemotherapy response rates in patients (Table 1). Consistent with protein data, NFIB mRNA was significantly upregulated in tumor tissues compared to matched paracancerous samples (Figure 1C).

**TABLE 1:** Correlations between NFIB expression and clinical/pathological features in SCLC.

Clinicopathologic features		Total, *n*	%	NFIB low		NFIB high		*p*
				*n*	%	*n*	%	
Gender	Male	74	62.2	15	20.2	59	79.8	0.738
	Female	45	37.8	8	17.8	37	82.2	
Age	≤ 60	67	56.3	9	13.4	58	86.6	0.065
	> 60	52	43.7	14	26.9	38	73.1	
Tobacco status	Non‐smoker	13	10.9	2	15.4	11	84.6	0.642
	Former smoker	23	19.3	6	26.1	17	73.9	
	Active smoker	83	69.8	15	18.1	68	81.9	
Histology type	Pure SCLC	106	89.1	18	17	88	83	0.128
	Mixed SCLC	13	10.9	5	38.5	8	61.5	
Tumor size (cm)	≤ 3	75	63.1	17	22.7	58	77.3	0.336
	> 3	44	36.9	6	13.6	38	86.4	
Syn	Negative	17	14.3	15	88.2	2	11.8	< 0.001
	Positive	102	85.7	8	7.8	94	92.2	
CgA	Negative	35	29.4	19	54.3	16	45.7	< 0.001
	Positive	84	70.6	4	4.8	80	95.2	
Ki‐67	Negative	21	17.6	13	61.9	8	38.1	< 0.001
	Positive	98	82.4	10	10.2	88	89.8	
Lymph node metastasis	Negative	61	51.3	17	27.9	44	72.1	0.020
	Positive	58	48.7	6	10.3	52	89.7	
Distant metastasis	Negative	105	88.2	23	21.9	82	78.1	0.041
	Positive	14	11.8	0	0	14	100	
TNM staging	I	52	43.7	15	28.8	37	71.2	0.006
	II	33	27.7	6	18.2	27	81.8	
	III	20	16.8	2	10	18	90	
	IV	14	11.8	0	0	14	100	
RECIST	CR	12	10.1	3	25	9	75	0.026
	PR	53	44.5	4	7.5	49	92.5	
	SD	36	30.3	14	38.9	22	61.1	
	PD	18	15.1	2	11.1	16	88.9	

*Note:* Data were expressed as *n* (%). *p* < 0.05 was considered statistically significant.

Abbreviations: CR means complete response; PD means progressive disease; PR means partial response; RECIST means response evaluation criteria in solid tumors; SCLC means small cell lung cancer; SD means stable disease.

### 3.2 Analysis of SCLC Cell Lines

NFIB protein expression varied across SCLC cell lines. Levels were significantly higher in H69, H209, and H446 cells compared to SHP77, H2227, and H524 cells, with H446 showing the highest and H2227 the lowest expression (Figure S1B,C). Based on this profile, we selected the H446 line (high NFIB) for knockdown experiments, the H2227 line (low NFIB) for overexpression studies, and the H209 line (moderate NFIB) for rescue experiments.

In human SCLC cells, we evaluated the role of NFIB in tumorigenesis and progression using shRNA‐mediated knockdown and quantified its biological effects on key oncogenic phenotypes. Knockdown of NFIB significantly increased apoptosis and decreased proliferation (Figure 1D,F), whereas NFIB overexpression suppressed apoptosis and promoted proliferation (Figure 1E,G). Notably, NFIB knockdown preferentially enhanced early apoptosis in tumor cells, with a more modest effect on late‐stage apoptosis. Furthermore, transwell assays demonstrated that NFIB knockdown impaired cellular invasion and migration, while NFIB overexpression enhanced these malignant phenotypes (Figure S1). Taken together, findings from both clinical specimens and cellular models substantiate the oncogenic role of NFIB in SCLC.

### 3.3 NFIB Binds to and Represses the Pro‐Apoptotic Genes BAK and BIK

**FIGURE 2:**
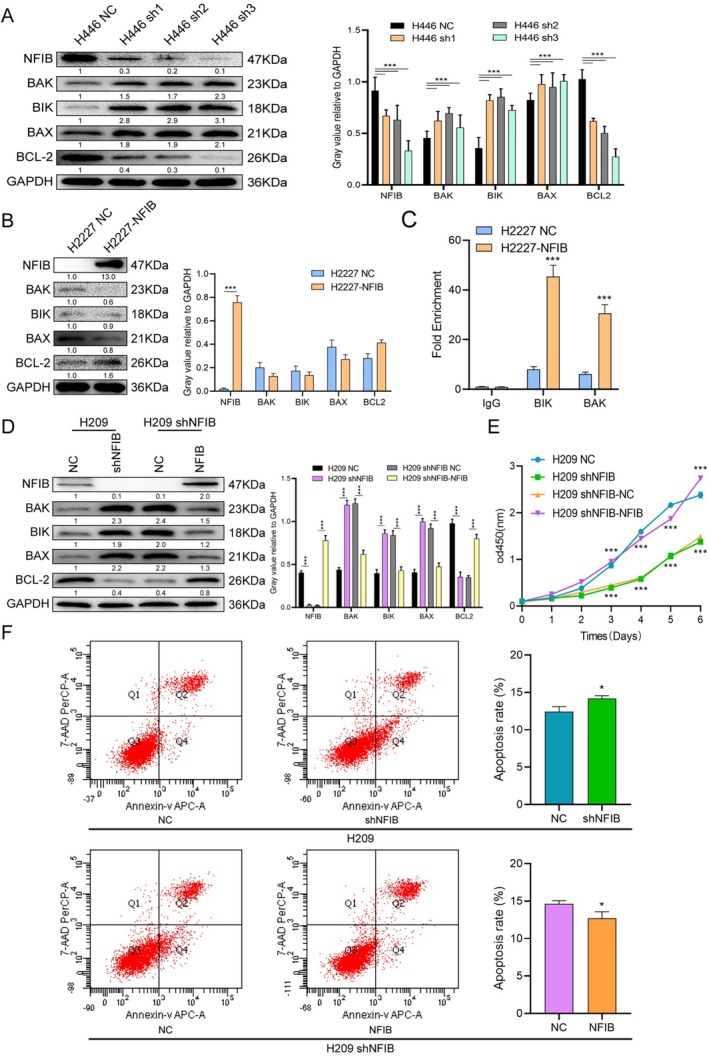
NFIB regulates pro‐apoptotic factors in vitro. (A and B) Western blot analysis was performed to determine the relative levels of various BCL‐2 family proteins involved in apoptosis following NFIB knockdown. GAPDH was used as a loading control. (C) Quantitative analysis of NFIB binding was conducted using ChIP‐PCR. The precipitated DNA from the BAK and BIK gene promoter regions was quantified. In H2227 cells, chromatin was immunoprecipitated using an NFIB antibody and then amplified by PCR. Rabbit IgG antibody and a GAPDH primer were used as a negative control. (D‐F) Western blotting (D), CCK‐8 assay (E), and flow cytometry (F) were performed to assess the role of NFIB in apoptosis induction in H209 cells. H209 cells were transduced with lentivirus to first knock down NFIB (denoted as H209 shNFIB), followed by subsequent NFIB overexpression (denoted as H209 shNFIB‐NFIB). Representative results from three independent experiments performed in triplicate are shown as mean ± SEM. *p < 0.05; **p < 0.01; ***p < 0.001 by *t* test.

To further investigate the role of NFIB in apoptosis regulation, we examined the expression of key apoptosis‐related proteins by Western blotting. Knockdown of NFIB significantly upregulated the pro‐apoptotic factors BIK and BAK and reduced the BCL‐2/BAX ratio from 1.25 to 0.51 (Figure 2A). Conversely, NFIB overexpression only slightly downregulated BIK and BAK while increasing the BCL‐2/BAX ratio from 0.77 to 1.48 (Figure 2B). In rescue experiments using H209 cells, re‐expression of NFIB reversed these apoptotic changes (Figure 2D–F), confirming that NFIB modulates the BCL‐2 family. ChIP‐PCR in NFIB‐overexpressing H2227 cells showed pronounced enrichment of NFIB at the promoters of BAK and BIK compared with vector controls (Figure 2C). These data indicate that NFIB directly binds to the promoter regions of BAK and BIK, leading to transcriptional repression and reduced protein expression. This mechanism explains why NFIB inhibition markedly upregulates BIK and BAK and lowers the BCL‐2/BAX ratio (Figure 2A). In summary, knockdown of NFIB in SCLC cells upregulates the key pro‐apoptotic regulators BAK and BIK, thereby promoting apoptosis. Our findings establish that NFIB supports SCLC cell survival primarily through the suppression of apoptosis.

### 3.4 NFIB Modulates Chemoresistance via Notch Pathway Regulation

**FIGURE 3:**
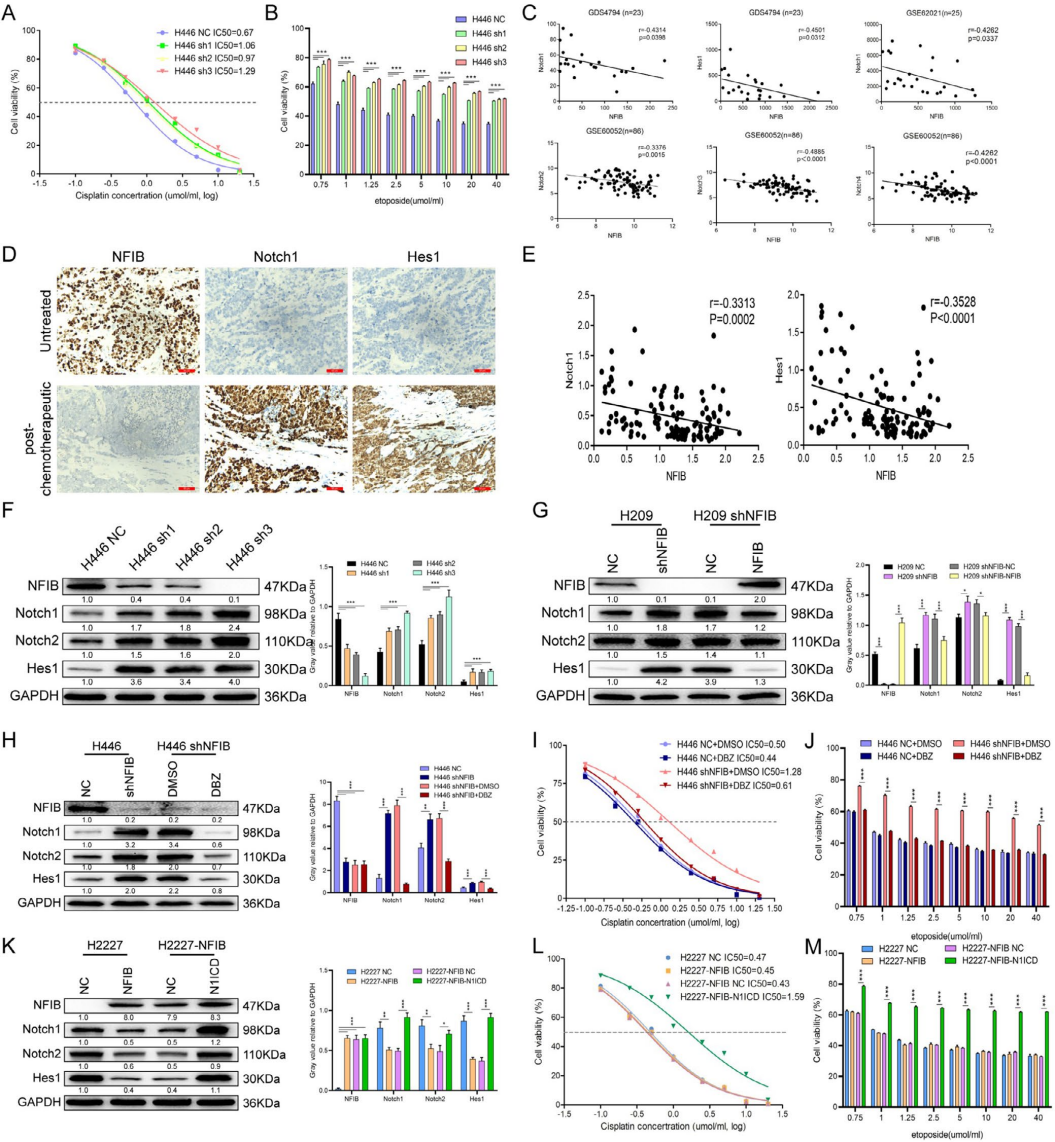
NFIB knockdown promotes chemoresistance by affecting Notch1. (A and B) Dose–response curves showing the relative viability of H446 cells with NFIB knockdown compared to control cells (NC) following 48‐h treatment with increasing concentrations of cisplatin (A) and etoposide (B). (C) Correlation analysis between NFIB and Notch family gene expression in microarray and RNA‐seq datasets from SCLC tissues. Analyses of the GDS4794 (*n* = 23), GSE62021 (*n* = 25), and GSE60052 (*n* = 86) datasets are shown. Pearson correlation coefficients (*r*) and corresponding *p* values are indicated. (D) Representative immunohistochemical staining of NFIB, Notch1, and Hes1 in chemotherapy‐treated versus chemotherapy‐naive SCLC patient samples. (E) Correlation analysis between Notch1, Hes1, and NFIB expression in SCLC tissues (*n* = 119) as assessed by IHC. Pearson correlation coefficients (*r*) and *p* values are indicated. (F) Western blot analysis of NFIB and Notch pathway components (Notch1, Notch2, Hes1) in H446 cells transduced with NFIB‐targeting shRNA. GAPDH was used as a loading control. (G) Western blot analysis of NFIB and Notch pathway proteins in H209 NC cells, H209 shNFIB cells, and the rescued H209 shNFIB‐NFIB cells. (H‐J) H446 NC and shNFIB cells were treated with either DMSO (control) or the DBZ. Protein levels of NFIB and Notch pathway components were assessed by Western blotting (H). Cell viability was measured by CCK‐8 assay after 48 h treatment with the indicated concentrations of cisplatin (I) and etoposide (J). (K) Western blot analysis of H2227 cells stably overexpressing NFIB following retroviral transduction with N1ICD (Notch1 intracellular domain) or an empty vector control. (L and M) Cell viability was assessed by CCK‐8 assay after 48 h treatment with increasing concentrations of cisplatin (L) and etoposide (M). Data are represented as mean ± SEM of three independent experiments. ***p < 0.001 by *t* test.

To determine whether NFIB influences the acquisition of chemoresistance in SCLC, we utilized standard chemotherapeutic agents for this disease—cisplatin and etoposide—both of which are commonly used in clinical practice yet frequently lead to rapid relapse and poor survival [23]. Upon treatment with etoposide or cisplatin, NFIB‐knockdown cells exhibited greater viability compared with control cells, indicating enhanced chemoresistance (Figure 3A,B). To investigate the underlying mechanism, we analyzed multiple SCLC patient datasets (GDS4794, GSE62021, and GSE60052) to identify genes correlated with chemoresistance (Figure 3C). Intriguingly, NFIB expression showed a negative correlation with Notch signaling activity, a pathway previously implicated in chemoresistance and tumor heterogeneity [24, 25]. Hes1, a downstream target of the Notch signaling pathway [11], has been demonstrated to mediate the acquisition of chemotherapy resistance in SCLC cells and serves as an adverse prognostic factor for both overall survival and progression‐free survival in SCLC patients [13].

Consistent with this, studies in SCLC mouse models have shown that cisplatin treatment expands the population of tumor cells with active Notch signaling, as evidenced by an increased frequency of Hes1‐positive cells [13]. This observation led us to hypothesize that Notch signaling activity is low in NFIB‐high SCLC contexts and that reducing NFIB expression would activate Notch1, thereby elevating Hes1. To test this, we examined the relationship between NFIB and Notch1 in SCLC patient samples using IHC. A clear pattern emerged: untreated tumor tissues showed high NFIB expression but low levels of Notch1 and Hes1 (Figure 3D). In contrast, post‐chemotherapy samples displayed markedly reduced NFIB alongside increased Notch1 and Hes1 (Figure 3D). NFIB expression showed a significant inverse correlation with the levels of both Notch1 and Hes1 (Figure 3E). Functionally, knockdown of NFIB notably increased the activity of Notch1 and Notch2, which was reflected by elevated Hes1 expression (Figure 3F). Conversely, NFIB overexpression reduced Notch1 and Notch2 levels, leading to decreased Hes1 expression (Figure 3K). These opposing effects indicate that NFIB acts as a negative regulator of the Notch signaling pathway. Rescue experiments in H209 cells further supported this mechanism: re‐expression of NFIB strongly inhibited Notch1 and Notch2, lowered their downstream target Hes1, and effectively suppressed pathway activity (Figure 3G). Together, these findings confirm that NFIB serves as a key upstream transcriptional repressor in SCLC cells.

To investigate whether NFIB regulates Notch signaling, we conducted a series of experiments. Treatment of H446‐shNFIB cells with the *γ*‐secretase inhibitor DBZ [26] significantly suppressed the expression of Notch1 and Notch2, along with their downstream target Hes1 (Figure 3H). Consistent with this, pharmacological inhibition of Notch signaling effectively reversed chemotherapy resistance in these cells (Figure 3I,J). Activation of Notch1 via N1ICD overexpression in H2227‐NFIB cells increased endogenous Notch2 and restored Hes1 levels (Figure 3K). Although NFIB overexpression did not affect chemoresistance, adding Notch1 overexpression in these cells markedly reduced sensitivity to cisplatin and etoposide (Figure 3L,M). Consistent with this hypothesis, results from the H209 rescue experiment provided additional support (Figure S2A–F). In contrast, DBZ alone failed to improve chemotherapy sensitivity in NFIB‐high H446 cells (Figure S3A,B). Although N1ICD overexpression alone in H2227 cells reduced chemotherapy sensitivity, the effect was stronger in H2227‐NFIB cells (Figure S3C,D). These results collectively indicate that NFIB inhibition relieves its direct suppression of Notch1, leading to upregulated Notch1 expression and subsequent activation of the downstream resistance mediator Hes1 in SCLC [11].

### 3.5 NFIB Directly Represses Notch1 Expression in SCLC

**FIGURE 4:**
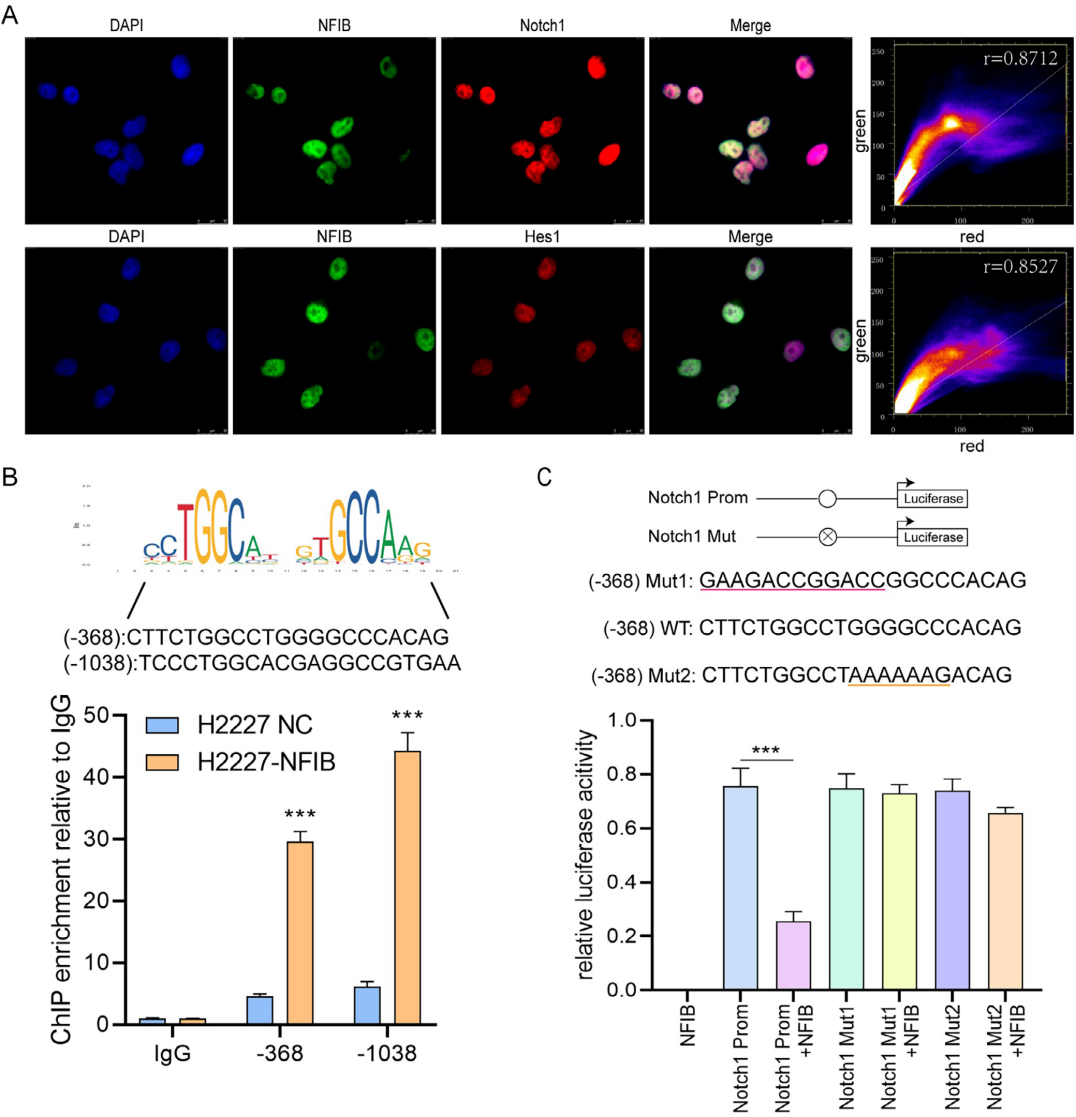
NFIB directly binds to the Notch1 promoter and represses its expression in SCLC. IF analysis of H209 cells showing subcellular localization of NFIB (red), Notch1 (green), and Hes1 (green). Nuclei were counterstained with DAPI. (B) ChIP‐PCR analysis of NFIB binding to the Notch1 promoter. Schematic shows the locations of predicted NFIB binding sites and their sequences. Rabbit IgG and GAPDH primers were used as negative controls. (C) Luciferase reporter assay in H2227 cells co‐transfected with a Notch1 promoter construct and either an NFIB‐expressing plasmid or empty vector (left panel). Luciferase activity was also measured in cells co‐transfected with NFIB‐expressing plasmid and either wild‐type or mutated (Mut1, Mut2) Notch1 promoter constructs (right panel). Promoter activity is expressed relative to control. Data represent mean ± SEM from three independent experiments performed in triplicate. ***p < 0.001 by *t* test.

To test this hypothesis, we first assessed the spatial relationship between NFIB, NOTCH1, and HES1 via IF. The results revealed clear nuclear co‐localization of these proteins in SCLC cells, indicating potential functional interactions (Figure 4A). Furthermore, analysis using the JASPAR database predicted NFIB binding sites within the NOTCH1 promoter region (Figure 4B). To corroborate this, we performed ChIP assays in H2227‐NFIB cells targeting the NOTCH1 promoter. Significant NFIB binding enrichment was detected at two regions: −1038 to −1058 bp and −368 to −388 bp (Figure 4B). Comparative analysis indicated that the−368 site was more evolutionarily conserved. Therefore, we selected the −368 region for functional validation using a luciferase reporter assay. We generated a mutant promoter (Mut) in which the predicted NFIB consensus sites within this region were specifically disrupted. In H2227‐NFIB overexpression lines, the mutant promoter exhibited significantly reduced responsiveness to NFIB compared to the wild‐type construct. Conversely, the wild‐type proximal NOTCH1 promoter showed a marked decrease in luciferase activity upon NFIB cotransfection (Figure 4C). These results demonstrate that NFIB directly occupies the endogenous NOTCH1 promoter and represses its transcriptional activity.

### 3.6 NFIB Knockdown Accelerates Phenotypic Transformation via Notch Activation

**FIGURE 5:**
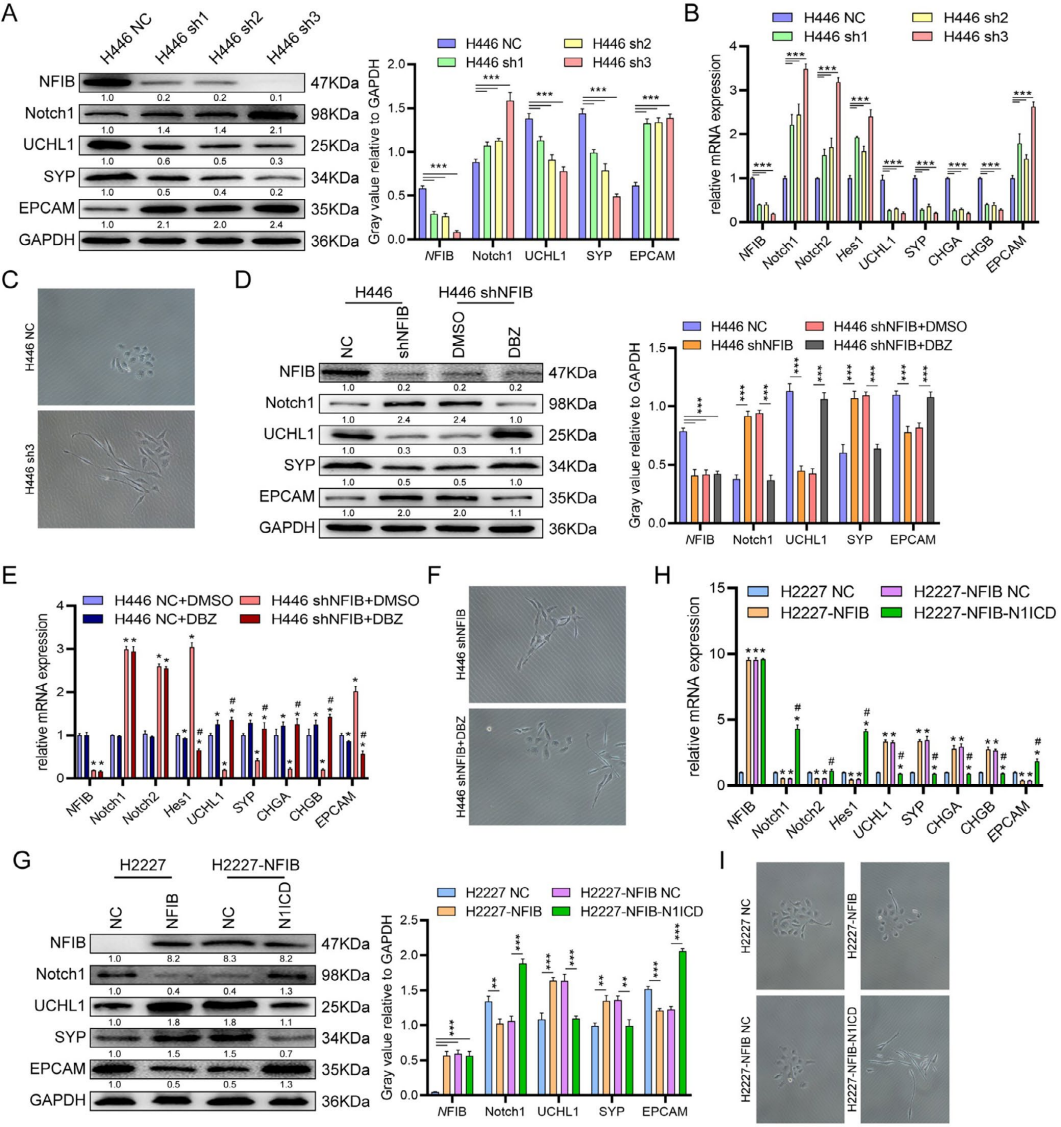
NFIB knockdown promotes cellular phenotypic transformation via Notch activation. (B) Western blotting (A) and qRT‐PCR (B) were used to assess the expression of NE markers and Notch pathway genes in H446 cells transduced with NFIB‐targeting shRNA. ***p < 0.0005. (C) Representative microscopy images showing morphological changes in H446 cells after NFIB knockdown. Scale bars, 50 μm. (D‐E) DBZ‐treated H446 shNFIB cells were analyzed by Western blotting (D) and qRT‐PCR (E) to evaluate expression of Notch pathway and NE marker genes. *p < 0.01 versus H446 NC group; ^#^ *p* < 0.01 versus H446 shNFIB+DMSO group. (F) Representative images of H446 shNFIB cells following DBZ treatment. (G‐I) Western blotting (G), qRT‐PCR (H), and representative microscopy images (I) of H2227 cells overexpressing NFIB after subsequent transduction with N1ICD. *p < 0.01 versus H2227 NC group; ^#^ *p* < 0.01 versus. H2227‐NFIB NC group. Data are represented as the mean ± SEM of the three independent experiments; the *p* value was determined by *t* test.

Previous studies have established that Notch1 and Hes1 play key roles in maintaining the balance between NE and non‐NE phenotypes [6, 13], as well as in promoting the transition from NE to non‐NE states [13]. Thus, we proposed that NFIB influences SCLC chemoresistance by facilitating a Notch‐driven phenotypic switch. As hypothesized, NFIB knockdown reduced the expression of neuroendocrine markers (UCHL1, SYP, CALCA, CHGA, CHGB) while increasing the epithelial marker EPCAM (Figure 5A,B, Figure S2C). Concurrently, SCLC cell lines shifted from growing as floating neuroendocrine clusters to adherent monolayers, reflecting a clear change in differentiation state (Figure 5C). Following DBZ treatment, the expression profile was partially restored; NE markers increased toward baseline (though still slightly suppressed), and EPCAM expression was modestly above control levels (Figure 5D,E). Correspondingly, cell morphology reverted toward a small, round shape—typical of neuroendocrine cells—from an elongated spindle shape (Figure 5F). Conversely, NFIB overexpression in SCLC cells increased NE marker expression and decreased EPCAM. Notably, co‐overexpression of N1ICD restored the marker profile, reducing NE markers to near baseline and raising EPCAM above baseline levels (Figure 5G,H, Figure S2F). In parallel, the cellular morphology transitioned from oval to short bipolar after NFIB overexpression, and back to oval following N1ICD induction (Figure 5I). In summary, NFIB knockdown triggered a reversible, Notch‐dependent phenotypic shift—marked by reduced NE markers, elevated EPCAM, and altered morphology—that could be partially reversed by NFIB re‐expression or Notch suppression. Nevertheless, this phenotypic plasticity was not uniformly present across all cell populations. Colony formation assays showed that NFIB silenced (H446 shNFIB) and NFIB‐overexpressing (H2227 NFIB) cell lines both exhibited altered colony‐forming capacities, further confirming the associated phenotypic changes (Figure S2G). Furthermore, in clinical SCLC tissues, NFIB expression showed positive correlations with levels of SYP, CHGA, and KI‐67 (Figure S2H, Table 1). In summary, our work demonstrates that NFIB constrains Notch signaling to regulate NE‐to‐non‐NE phenotypic plasticity in SCLC, a mechanism linked to chemoresistance.

Inhibition of NFIB led to Notch1 upregulation and a concomitant decrease in ASCL1, a master regulator of NE differentiation [14]. The downregulation of ASCL1 at both protein and mRNA levels was dependent on elevated Notch1, since DBZ treatment rescued ASCL1 expression to above‐baseline levels (Figure S4A,B). Conversely, in H2227 cells overexpressing NFIB (where Notch1 is low), ASCL1 expression was elevated. Re‐activation of Notch1 via N1ICD overexpression in these cells potently suppressed ASCL1 (Figure S4C,D). Rescue experiments in NFIB‐knockdown H209 cells showed that NFIB re‐expression lowered Notch1 and correspondingly elevated ASCL1 (Figure S4E,F). Furthermore, analysis of public datasets (GDS4794, GSE62021) revealed a positive correlation between NFIB and ASCL1 mRNA levels (Figure S4G). These results collectively propose a coordinated regulatory network, wherein NFIB concurrently regulates both positive and negative drivers of neuroendocrine differentiation to precisely control the cellular phenotype in SCLC.

**FIGURE 6:**
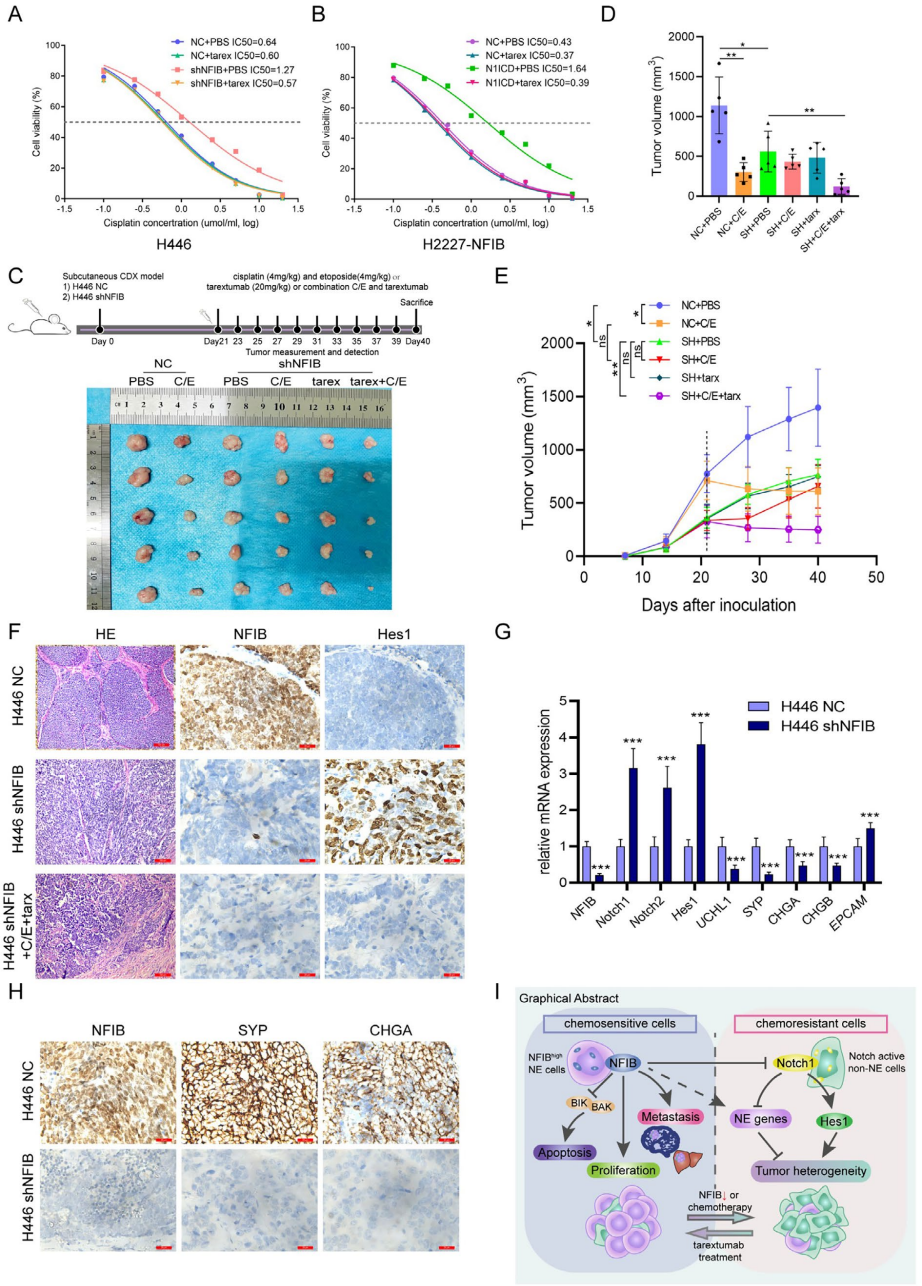
Combination therapy with chemotherapy and a Notch inhibitor reduces SCLC chemoresistance in vitro and in vivo. The relative cell viability of H446 shNFIB cells was assessed following combined treatment with cisplatin and tarextumab (tarex) for 48 h. (B) Viability of H2227‐NFIB cells stably overexpressing N1ICD was measured after 48 h treatment with tarextumab and cisplatin. (C) *Xenografts* were established by inoculating nude mice (*n* = 5 per group) with H446 cells transduced with NFIB‐knockdown or empty vector. Drug administration schedule was recorded, and tumor images were captured after 40 days. (D) Final tumor volumes were measured upon excision from each group. (E) Tumor growth curves illustrate changes in tumor volume across experimental groups. (F) Representative IHC staining of Hes1 and NFIB in excised tumor tissues. Scale bars = 25 μm. (G and H) NE gene expression in tumor tissues was assessed by qRT‐PCR (G) and IHC staining (H). (I) Schematic model summarizing the major findings of the study. Data represent mean ± SEM from three independent experiments performed in triplicate, ns, not significant. *p < 0.05; **p < 0.01; ***p < 0.001 by *t* test.

To evaluate the therapeutic potential of targeting Notch in NFIB‐associated chemoresistance, we treated NFIB‐knockdown SCLC cells with the Notch antagonist tarextumab in combination with cisplatin. Western blot analysis confirmed that tarextumab effectively reduced Notch1 and Notch2 protein levels (Figure S3E). Interestingly, tarextumab treatment rescued the cisplatin resistance induced by NFIB knockdown (Figure 6A, Figure S3F,G). In H2227 cells overexpressing NFIB, tarextumab alone had little effect on chemoresistance (Figure 6B). However, subsequent N1ICD overexpression in these cells substantially increased resistance, which was effectively reversed by co‐treatment with tarextumab (Figure 6B, Figure S3H,I). Taken together, these results indicate that NFIB promotes chemoresistance primarily via Notch1 activation, an effect that can be blocked by the Notch antagonist tarextumab.

We assessed the therapeutic potential of NFIB targeting using mouse *xenograft* models. Although tumors derived from NFIB‐knockdown cells were smaller, they showed greater resistance to chemotherapy than wild‐type tumors. Strikingly, the triple combination of etoposide, cisplatin, and tarextumab significantly inhibited tumor growth in H446 shNFIB models, while neither chemotherapy alone nor tarextumab alone was effective (Figure 6C–E). Consistent with in vitro observations, the combination treatment also reduced Hes1 and NFIB levels in vivo (Figure 6F). Multipoint tumor sampling further demonstrated that NFIB knockdown downregulated neuroendocrine genes and increased epithelial markers (Figure 6G,H). These results collectively demonstrate that NFIB depletion promotes chemoresistance by alleviating Notch1‐mediated suppression and driving phenotypic plasticity. The addition of tarextumab reverses this resistance, explaining why the triple combination (etoposide, cisplatin, and tarextumab) effectively restrained tumor growth and blocked chemoresistance development.

## 4 Discussion

SCLC is associated with a dismal prognosis, marked by rapid relapse and inherent chemoresistance. The paucity of efficacious second‐line treatment options underscores an unmet clinical need for innovative therapeutic strategies [27, 28, 29]. Emerging preclinical and clinical evidence supports the rationale for combinatorial regimens that integrate chemotherapy with pathway‐specific targeted agents—including EZH2 epigenetic modifiers, PD‐L1 immune checkpoint inhibitors, and CHK1 DNA‐damage response regulators [30, 31, 32]. While existing evidence confirms that targeting these genes modifies baseline chemosensitivity in SCLC, deciphering the underlying molecular pathways remains critical for therapeutic optimization [33, 34].

NFIB has been previously characterized as an oncogenic factor in SCLC [18, 19]. Our data confirm its frequent upregulation in SCLC (notably in brain metastases), where its expression correlates with lymph node involvement, distant metastasis, and advanced disease stage—collectively underscoring its key role in tumor progression and metastasis [17]. Consistent with this role, we found that high NFIB expression promotes tumor cell invasion, motility, proliferation, and apoptosis resistance (Figure S1). Interestingly, NFIB—a gene critical for brain development and neuronal migration [20]—also modulates neuronal‐associated genes, which aligns with the positive correlation we observed between NFIB and neuroendocrine differentiation markers in SCLC cell lines. This functional profile is further supported by reports of elevated NFIB in human primitive neuroectodermal tumors and murine SCLC models [21].

However, SCLC tumors exhibit profound intratumoral heterogeneity. While some studies suggest that NFIB‐expressing tumors may respond better to treatment [35], its role in chemoresistance appears complex. Our investigation revealed that NFIB modulates chemoresistance distinctly from its pro‐metastatic function, primarily through regulating the Notch1‐Hes1 axis—a key pathway in drug resistance [13]. Our mechanistic studies show that Notch1 inhibition reverses NFIB depletion‐driven drug resistance, while NFIB overexpression attenuates Notch1‐mediated chemoresistance. The Notch pathway, a key regulator of cell fate, exhibits context‐dependent roles in cancer [4, 36, 37]. Despite its established role as a tumor suppressor and master regulator of neuroendocrine (NE) differentiation, emerging evidence implicates Notch in SCLC chemoresistance by facilitating the NE‐to‐non‐NE phenotypic switch [13]. Mechanistically, we establish that NFIB binds directly to the NOTCH1 promoter and represses HES1 expression, defining a functional NFIB‐NOTCH1 axis. This provides a mechanism for NFIB‐mediated chemoresistance; however, clinical correlations are complex. Strikingly, NFIB depletion potently enhanced chemoresistance, whereas its overexpression only modestly reduced it, indicating that NFIB's effect is critically dependent on NOTCH1 activity levels. These findings highlight the need for further work to delineate the functional interactions between NFIB and NOTCH pathway effectors. Reported prognostic associations of Notch1 in SCLC appear stage‐dependent: low Notch1 correlates with better survival in advanced disease [38], whereas Notch pathway deficiency is linked to poor prognosis in early‐stage cohorts [37]. Our analysis, confined to advanced‐stage patients, aligns with the former observation. A limitation of the present study is the absence of correlation between NFIB expression and survival outcomes. Future analyses integrating comprehensive survival data are needed to definitively establish the clinical relevance of the NFIB‐Notch signaling axis.

ASCL1 is a known downstream effector of Notch1 [39] and a critical NE lineage transcription factor in SCLC [40, 41, 42]. Paradoxically, our data show that NFIB elevates ASCL1 levels despite suppressing its putative regulator, Notch1 (Figure S4). This contrasts with independent evidence suggesting a cooperative complex between ASCL1 and NFIB^42^. Functional assays confirmed that ASCL1 is negatively regulated by Notch activity (Figure S4A–F). We therefore propose a model wherein NFIB promotes ASCL1 via a direct (Notch1‐independent) mechanism, while its concurrent repression of Notch1 signaling further modulates ASCL1 expression, resulting in a complex regulatory outcome. Collectively, our work positions NFIB as a pivotal upstream node that sustains the NE phenotype by dually repressing Notch1 and enhancing ASCL1. We propose that this coordinated regulation maintains the high ASCL1 activity necessary for SCLC cell survival. The clinical correlation between NFIB and ASCL1 further supports this model. However, key questions remain: whether NFIB's effect on ASCL1 is mediated entirely through Notch1 inhibition, or involves direct protein interactions, and the exact molecular nature of their functional relationship require elucidation.

The paucity of stromal cells accounts for the pronounced microenvironmental heterogeneity in SCLC [43, 44]. These tumors also exhibit intrinsic cellular heterogeneity, comprising distinct subpopulations with differing chemosensitivities [35], which critically shapes responses to cisplatin‐based therapy [11, 23]. Our work demonstrates that NFIB‐enriched SCLC cells possess heightened proliferative, migratory, and invasive capacities, aligned with a strong neuroendocrine (NE) phenotype. NFIB knockdown reverses this state, inducing a non‐NE transition coupled with Notch pathway activation—consistent with Notch's context‐dependent roles in cancer. These insights clarify NFIB's contribution to SCLC pathogenesis and chemoresistance, pointing to co‐targeting NFIB and Notch1 as a potential strategy to overcome intrinsic drug resistance and improve therapeutic outcomes.

To advance the therapeutic targeting of NFIB and Notch1, we investigated their inhibition in combination with standard chemotherapy. The anti‐Notch2/3 antibody tarextumab, evaluated in a Phase I trial for SCLC, showed gastrointestinal toxicity as the most common adverse event. Doses below 2.5 mg/kg weekly or 7.5 mg/kg every 2–3 weeks were generally well‐tolerated [45]. Notably, the combination of cisplatin, etoposide, and tarextumab proved feasible in patients with advanced SCLC and was associated with improved survival in those exhibiting high Notch target gene expression. Our preclinical in vitro and in vivo studies corroborate these findings, demonstrating synergistic anti‐tumor effects for this combination regimen.

To develop a personalized treatment strategy for SCLC, we investigated the therapeutic potential of co‐targeting NFIB and Notch1. Our findings indicate that combined inhibition of this axis may prevent the acquisition of chemoresistance, arguing for a systematic, pathway‐based approach over strategies focused on single genetic alterations. This approach entails a stage‐aware therapeutic paradigm. In early‐stage, highly invasive tumors with elevated NFIB, initial treatment would target NFIB. Concurrently, it is critical to anticipate and counteract the development of a post‐therapy resistant microenvironment, with a focus on mechanisms such as Notch pathway antagonism. Proactively addressing these resistance pathways from the outset is essential for achieving durable treatment outcomes. Further studies are warranted to validate the clinical relevance of the NFIB‐Notch1 axis and to translate this combinatorial strategy into improved therapies for patients with advanced solid tumors.

## Author Contributions

**Weixin Qin:** conceptualization, data curation, formal analysis, methodology, validation, writing – original draft, writing – review and editing, investigation. **Ziyan Wang:** conceptualization, data curation, methodology, project administration, validation, writing – review and editing, investigation. **Huilei Qiu:** data curation, investigation. **Hongxue Meng:** conceptualization. **Jingshu Geng:** conceptualization, formal analysis, project administration, resources, writing – review and editing.

## Funding

The authors have nothing to report.

## Ethics Statement

This study was approved by the Ethics Committee of Harbin Medical University (Approval No. IRB5012723).

## Conflicts of Interest

The authors declare no conflicts of interest.

## Supporting information


**Figure S1:** NFIB is an oncogenic gene in SCLC.
**Figure S2:** NFIB inhibits the Notch signaling pathway and is associated with neuroendocrine differentiation.
**Figure S3:** Inhibition of Notch1/2 signaling reduces the Notch‐active cell population and enhances chemosensitivity in SCLC in combination with chemotherapy.
**Figure S4:** Knockdown of NFIB reduces ASCL1 expression through activation of NOTCH1 signaling.
**Table S1:** Antibodies.
**Table S2:** Primer sequences for qRT‐PCR.
**Table S3:** Cell line nomenclature and modification details.

## Data Availability

The data that supports the findings of this study are available in the Supporting Information of this article.
