# Supplementary material for: NFIB Regulates Chemoresistance in Small Cell Lung Cancer by Suppressing Notch Signaling Activity
Source: Cancer Rep (Hoboken). 2026 Apr 19;9(4):e70526. doi: 10.1002/cnr2.70526 (PMC13092339; doi:10.1002/cnr2.70526)
Supplement: Supplementary file 1 — Figure S1: NFIB is an oncogenic gene in SCLC. Figure S2: NFIB inhibits the Notch signaling pathway and is associated with neuroendocrine differentiation. Figure S3: Inhibition of Notch1/2 signaling reduces the Notch‐active cell population and enhances chemosensitivity in SCLC in combination with chemotherapy. Figure S4: Knockdown of NFIB reduces ASCL1 expression through activation of NOTCH1 signaling. Table S1: Antibodies. Table S2: Primer sequences for qRT‐PCR. Table S3: Cell line nomenclature and modification details. [file CNR2-9-e70526-s001.docx]

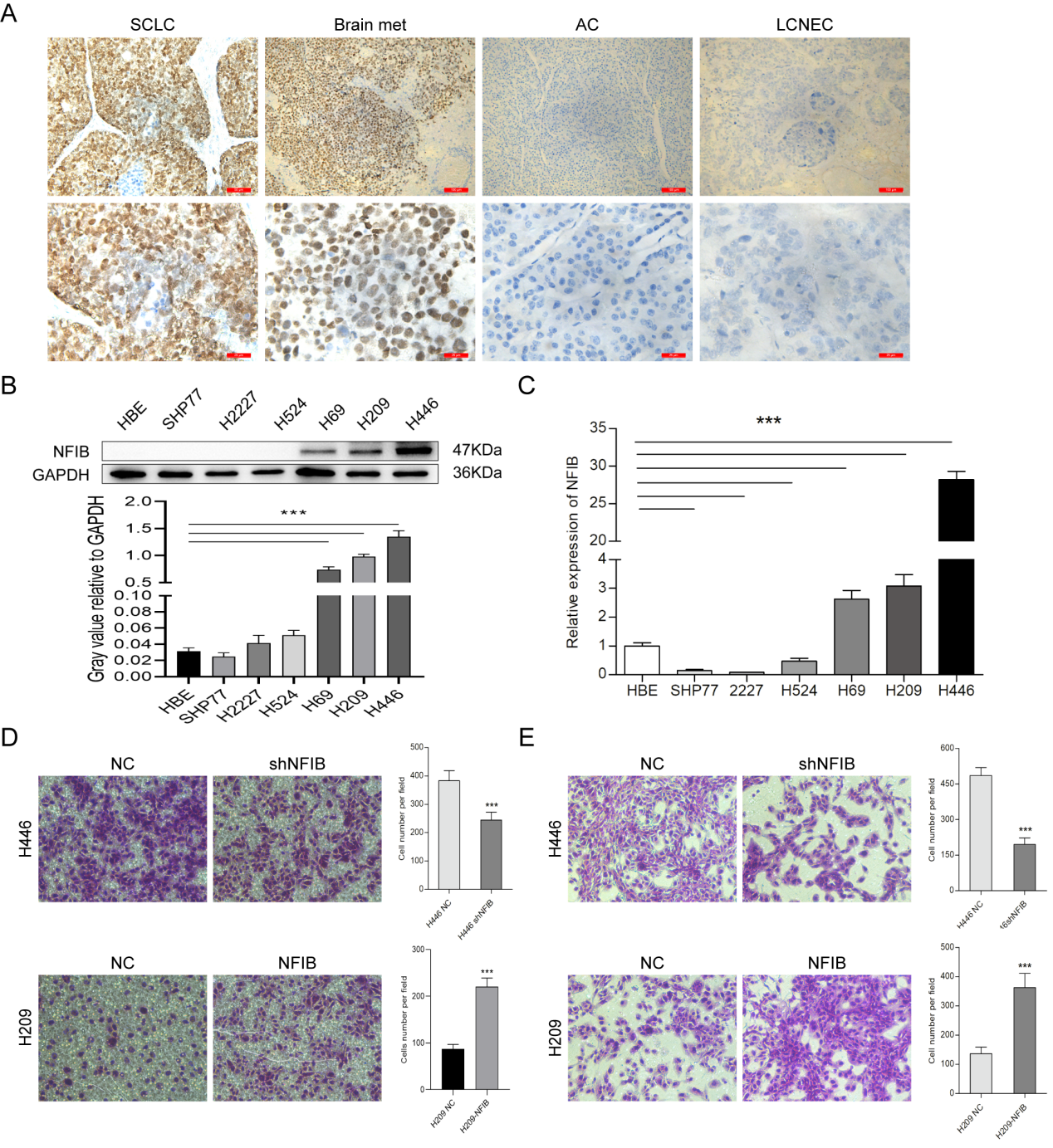


Figure S1. NFIB is an oncogenic gene in SCLC.

IHC staining of NFIB SCLC brain metastases, adenocarcinoma (AC), and large cell neuroendocrine carcinoma (LCNEC). (B and C) Western blotting (B) and quantitative RT-PCR (qRT-PCR) (C) analysis of NFIB expression. GAPDH was used as a loading control. (D and E) Transwell assays were performed to examine the migratory (D) and invasive (E) capacities of SCLC cells following NFIB knockdown or overexpression. Representative results in triplicate from 3 independent experiments are shown as mean ±SEM. ****P* <0.001 by t test.


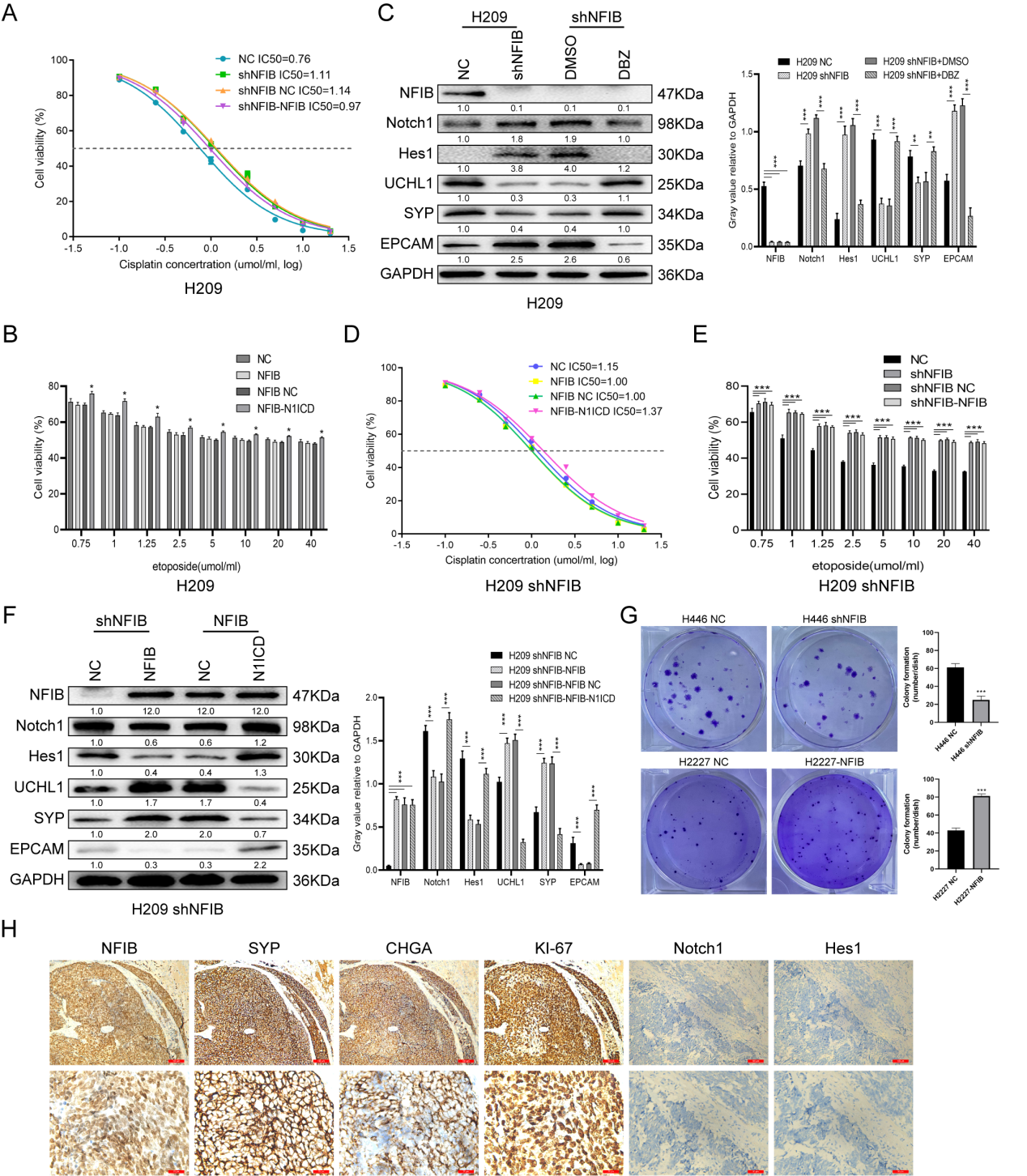


Figure S2. NFIB inhibits the Notch signaling pathway and is associated with neuroendocrine differentiation.

(A and B) Relative cell viability of H209 shNFIB, H209 shNFIB stably overexpressing NFIB (H209 shNFIB-NFIB), and control cells treated with the indicated concentrations of cisplatin (A) or etoposide (B) for 48 hours. (C) H209 NC and shNFIB cells were treated with DMSO (control) or DBZ; protein levels of NFIB, Notch pathway components, and GAPDH were analyzed by Western blot. (D-F) H209 shNFIB cells stably overexpressing NFIB were transduced with an N1ICD-encoding retrovirus or an empty vector control. Protein levels were evaluated (D), and cells were treated with different concentrations of cisplatin (E) or etoposide (F) for 48 hours. Cell viability was measured using the CCK-8 assay. (G) Colony formation assays were performed in both NFIB-knockdown(H446 shNIFB) and NFIB-overexpressing(H2227 NFIB) cell lines to determine functional consequences. (H) Representative immunohistochemical staining of NFIB, SYP, CHGA, KI-67, Notch1, and Hes1 in SCLC tissue samples. Data are representative of three independent experiments performed in triplicate and are shown as mean ± SEM. **P* < 0.05; ***P* < 0.01; ****P* <0.001 by t test.


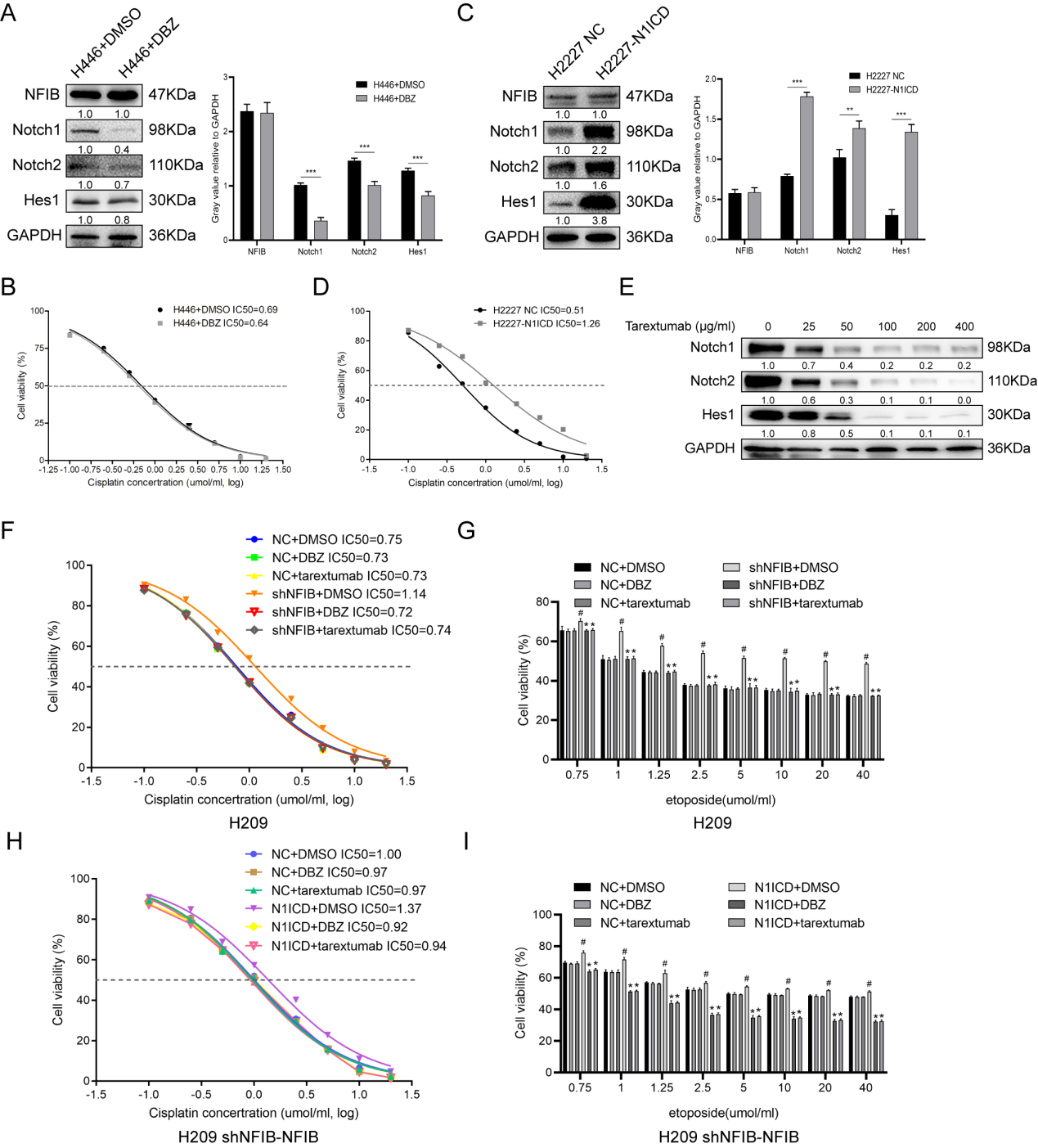


Figure S3. Inhibition of Notch1/2 signaling reduces the Notch-active cell population and enhances chemosensitivity in SCLC in combination with chemotherapy.

(A-B) H446 cells were treated with DMSO (control) or DBZ. Protein levels of NFIB, Notch pathway components, and GAPDH were analyzed by Western blotting (A). The relative viability of these cells treated with the indicated concentrations of cisplatin for 48 hours was assessed using a CCK-8 assay (B). (C-D) H2227 cells were transduced with an N1ICD-encoding retrovirus or an empty vector control. Protein levels were evaluated (C), and transduced cells were treated with different concentrations of cisplatin for 48 hours. Cell viability was measured using the CCK-8 assay (D). (E) Western blot analysis of Notch pathway components in H446 cells transduced with NFIB shRNA after treatment with tarextumab. (F-G) H209 NC and shNFIB cells were treated with DMSO, DBZ, or tarextumab in the presence of indicated concentrations of cisplatin (F) or etoposide (G) for 48 hours.#*P* < 0.01 vs. H209 NC; **P* < 0.01 vs. H209 NC treated with DBZ or tarextumab. (H-I) H209 shNFIB-NFIB cells transduced with an N1ICD-encoding retrovirus were treated with DMSO, DBZ, or tarextumab along with indicated concentrations of cisplatin (H) or etoposide (I) for 48 hours. #*P* < 0.01 vs. H209 shNFIB-NFIB NC; **P* < 0.01 vs. H209 shNFIB-NFIB NC treated with DBZ or tarextumab.Data are representative of three independent experiments performed in triplicate and are shown as mean ± SEM. **P* < 0.05; ***P* < 0.01; ****P* <0.001 by t test.


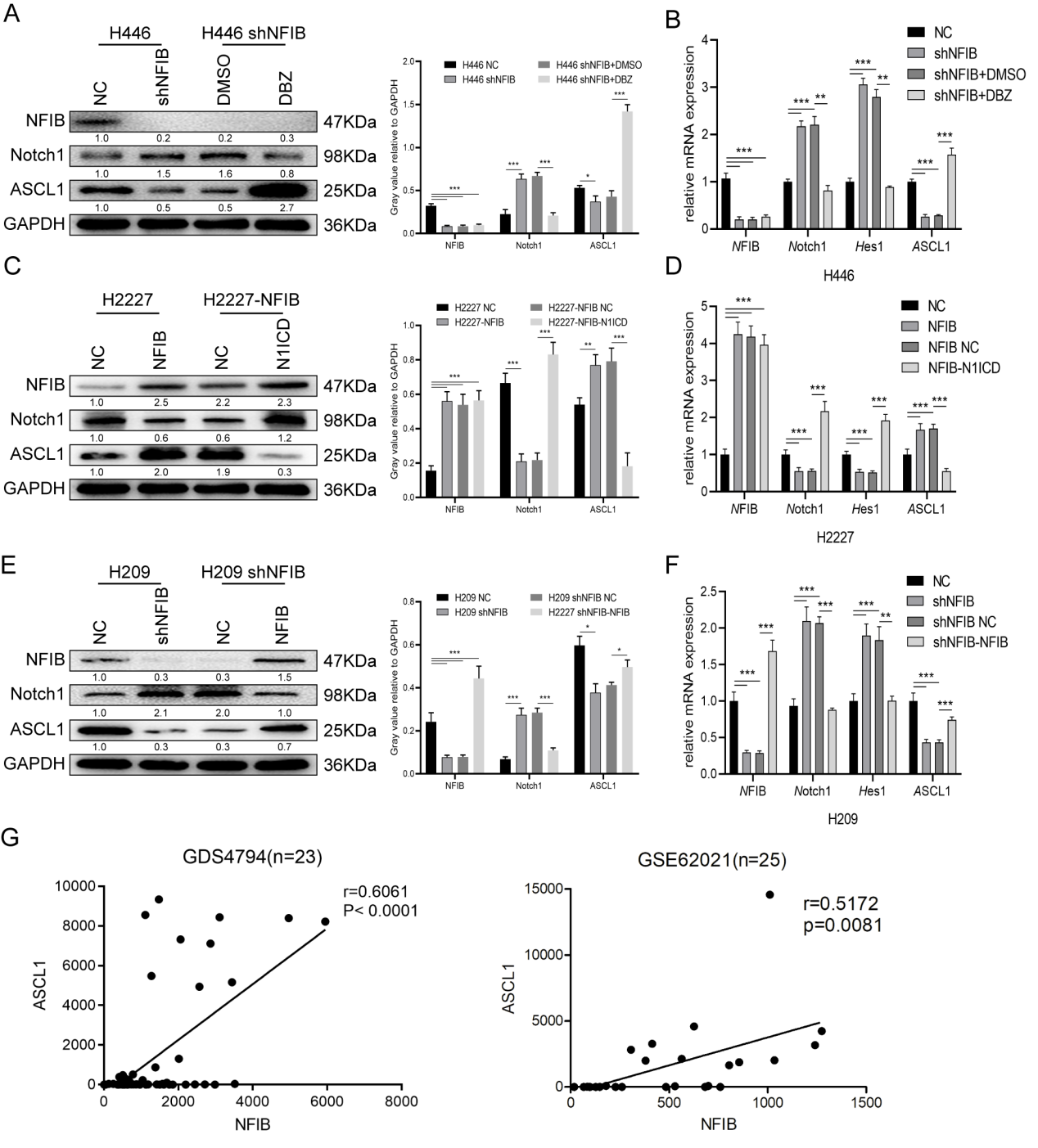
Figure S4. Knockdown of NFIB reduces ASCL1 expression through activation of NOTCH1 signaling.

(A-B) H446 NC and shNFIB cells were treated with DMSO or DBZ. Protein and mRNA expression levels of NFIB, NOTCH1, ASCL1, and GAPDH were analyzed by Western blotting (A) and qRT-PCR (B), respectively. (C–D) H2227 cells stably overexpressing NFIB were transduced with N1ICD retrovirus or empty vector. NFIB, NOTCH1, ASCL1, and GAPDH expression was evaluated by Western blotting (C) and qRT-PCR (D). (E–F) Protein (E) and mRNA (F) levels of NFIB, NOTCH1, ASCL1, and GAPDH were measured in H209 NC, H209 shNFIB, H209 shNFIB NC, and H209 shNFIB-NFIB rescue cells. (G) Correlation analysis between NFIB and ASCL1 expression in SCLC tissue datasets GDS4794 (n = 23) and GSE62021 (n = 25). Pearson correlation coefficient (r) and p-values are indicated. Data are presented as mean ± SEM from three independent experiments, each performed in triplicate. **P* < 0.05; ***P* < 0.01; ****P* <0.001 by t test.

Table S1. Antibodies.

| **Antibodies** | **Type** | **Catalog #** | **Application** |
| --- | --- | --- | --- |
| NFIB | rabbit polyclonal | ab186738, Abcam | Western blot,IF,IHC |
| NFIB | mouse monoclonal | NBP2-75589, Novus | IF |
| NOTCH1 | rabbit polyclonal | #3608, Cell signaling technology | Western blot,IF,IHC |
| Cleaved Notch1 (Val1744) | rabbit polyclonal | #4147, Cell signaling technology | Western blot |
| NOTCH2 | rabbit polyclonal | #5732, Cell signaling technology | Western blot |
| HES1 | rabbit polyclonal | ab71559, Abcam | Western blot,IF,IHC |
| Bax | rabbit polyclonal | #2772, Cell signaling technology | Western blot |
| Bcl2 | rabbit polyclonal | #3498, Cell signaling technology | Western blot |
| Bak | rabbit polyclonal | #6947, Cell signaling technology | Western blot |
| BIK | rabbit polyclonal | #4592, Cell signaling technology | Western blot |
| UCHL1 | rabbit polyclonal | #13179, Cell signaling technology | Western blot |
| EPCAM | rabbit polyclonal | #93790, Cell signaling technology | Western blot |
| ASCL1 | rabbit polyclonal | ab74065, Abcam | Western blot |
| GAPDH | mouse polyclonal | 10494-1-AP, Proteintech | Western blot |

Table S2: Primer sequences for qRT-PCR.

| **Target** | **Primer Sequence for SYBR Green RT-PCR(5'-3')** |
| --- | --- |
| NFIB | F: CAGTGGTCAAGTAGTAGGGAAAG |
|  | R: AGTGGATGTAGTGATGGGTTTAG |
| Notch1 | F: AAGTACTTCAGTGACGGCCA |
|  | R: GCCGTAGTAGGGGAAGATC |
| Notch2 | F: TTGTGACATAGCAGCCTCCA |
|  | R: CATTCTGGCAGGGCTGATTC |
| Hes1 | F: GTCAACACGACACCGGATAA |
|  | R: TTCAGCTGGCTCAGACTTTC |
| SYP | F: CGCCTTCATGTGGCTAGTT |
|  | R: CAGACAGGCATCTCCTTGATAAT |
| CHGA | F: GGGATACCGAGGTGATGAAATG |
|  | R: TCTCCTCGGAGTGTCTCAAA |
| CHGB | F: AGAAAGCAGGCTTCAGCTATAA |
|  | R: CTTCTCTCGGCTATGTGTCTTC |
| UCHL1 | F: TGCTGAACAAAGTGCTGTCC |
|  | R: CCGATTGTGCCACAGGAATT |
| EPCAM | F: CAGAAGGAGATCACAACGCG |
|  | R: TCCAGATCCAGTTGTTCCCC |
| ASCL1 | F: AACCGCGTCAAGTTGGT |
|  | R: CTCCACCTTACTCATCTTCTTGT |
| GAPDH | F: GGTGTGAACCATGAGAAGTATGA |
|  | R: GAGTCCTTCCACGATACCAAAG |
|  |  |
| **Target** | **ChIP qPCR primers(5'-3')** |
| Notch1 prmoter(-368) | F: CCGCAAAATGCTGAGGACTG |
|  | R: TGCCTGTGTGCCTCCATTG |
| Notch1 prmoter(-1038) | F: GCACTCACCACCCTGTGTT |
|  | R: GTTAAGCGCCAGGAGTCCAA |
| BIK prmoter | F: TCCAGTGTCCCCAAACGGTC |
|  | R: GGGGTCTCATGGGTCCGAAA |
| BAK prmoter | F: AGCTCTGCCTCCTGAGTTC |
|  | R: GAAACCCCGTCTCTACTA |

Table S3:Cell line nomenclature and modification details

| **Designation** | **Parental Line** | **Genetic Manipulation** | **Control** |
| --- | --- | --- | --- |
| **H446-shNFIB** | H446 | lentiviral NFIB knockdown | H446 NC |
| ****H2227-NFIB**** | H2227 | Lentiviral NFIB overexpression | H2227 NC |
| ****H2227-NFIB-N1ICD**** | H2227-NFIB | Lentiviral Notch1 overexpression | H2227-NFIB NC |
| ****H209-shNFIB**** | H209 | lentiviral NFIB knockdown | H209-NC |
| ****H209-shNFIB-NFIB**** | H209-shNFIB | Lentiviral NFIB overexpression | H209-shNFIB NC |
| ****H209-shNFIB-NFIB-N1ICD**** | H209-shNFIB-NFIB | Lentiviral Notch1 overexpression | H209-shNFIB-NFIB NC |
